# Preoperative serum immunoglobulin G and A antibodies to *Porphyromonas gingivalis* are potential serum biomarkers for the diagnosis and prognosis of esophageal squamous cell carcinoma

**DOI:** 10.1186/s12885-017-3905-1

**Published:** 2018-01-03

**Authors:** She-Gan Gao, Jun-Qiang Yang, Zhi-Kun Ma, Xiang Yuan, Chen Zhao, Guang-Chao Wang, Hua Wei, Xiao-Shan Feng, Yi-Jun Qi

**Affiliations:** 1 Henan Key Laboratory of Cancer Epigenetics; Cancer Hospital, The First Affiliated Hospital, College of Clinical Medicine, Medical College of Henan University of Science and Technology, Luoyang, Henan 471003 People’s Republic of China; 2 Department of Oral Mucosal Diseases, Shanghai Ninth People’s Hospital, Shanghai Jiao Tong University School of Medicine, Shanghai, 200011 People’s Republic of China; 3 Huaihe Hospital, Henan University, Kaifeng, Henan 475004 People’s Republic of China

**Keywords:** Esophageal squamous cell carcinoma, *Porphyromonas gingivalis*, Antibody, Immunoglobulin G/A, Diagnosis, Prognosis

## Abstract

**Background:**

The key-stone-pathogen, *Porphyromonas gingivalis* associates not only with periodontal diseases but with a variety of other chronic diseases such as cancer. We previously reported an association between the presence of *Porphyromonas gingivalis* in esophageal squamous cell carcinoma (ESCC) and its progression. We now report the diagnostic and prognostic potential of serum immunoglobulin G and A antibodies (IgG/A) against *Porphyromonas gingivalis* for ESCC.

**Methods:**

An enzyme-linked immunosorbent assay (ELISA) was used to determine the serum levels of *Porphyromonas gingivalis* IgG and IgA in 96 cases with ESCC, 50 cases with esophagitis and 80 healthy controls.

**Results:**

The median serum levels of IgG and IgA for *P. gingivalis* were significantly higher in ESCC patients than non-ESCC controls. *P. gingivalis* IgG and IgA in serum demonstrated sensitivities/specificities of 29.17%/96.90% and 52.10%/70.81%, respectively, and combination of IgG and IgA produced a sensitivity/specificity of 68.75%/68.46%. The diagnostic performance of serum *P. gingivalis* IgA for early ESCC was superior to that of IgG (54.54% vs. 20.45%). Furthermore, high serum levels of *P. gingivalis* IgG or IgA were associated with worse prognosis of ESCC patients, in particular for patients with stage 0-IIor negative lymphnode metastasis, and ESCC patients with high levels of both IgG and IgA had the worst prognosis. Multivariate analysis revealed that lymph node status, IgG and IgA were independent prognostic factors.

**Conclusions:**

The IgG and IgA for *P. gingivalis* are potential serum biomarkers for ESCC and combination of IgG and IgA improves the diagnostic and prognostic performance. Furthermore, serum *P. gingivalis* IgG and IgA can detect early stage ESCC.

**Electronic supplementary material:**

The online version of this article (10.1186/s12885-017-3905-1) contains supplementary material, which is available to authorized users.

## Background

Esophageal squamous cell carcinoma (ESCC) remains the predominant histological subtype of esophageal carcinoma and ranks as the fourth most common cancer in terms of both incidence and mortality in China [1, 2]. Although significant advances in diagnostic and therapeutic modalities have improved the prognosis of ESCC patients, the overall 5-year survival rate still ranges from 25% to 30%, mainly due to advanced stage at initial presentation [1, 3–7]. On the other hand, accurate staging and prognosis is difficult to assess at diagnosis, which hampers ESCC tailoring therapy, treatment efficiency and recurrence monitoring. It is, therefore, imperative to identify novel biomarkers for early detection, metastasis and recurrence to reduce ESCC-related morbidity and mortality.

A number of epidemiological and clinical studies have reported a positive association between the conditions of oral microbiome, periodontal disease or tooth loss and the progression of multiple cancers [8–25], and even gastric precancerous lesions [26, 27]. The oral microbiome inhabiting the oral cavity contains multiple species in a complex community that generally exist in a balanced immunoinflammatory state with the host [28]. Disruption of this equilibrium has deleterious effects on the mucosal lining, surrounding tissues and even distant organs and systems of human body through the combined effects of a dysbiotic microbial community and a dysregulated immune response [12, 13, 29]. *Porphyromonas gingivalis* has become regarded as a key-stone pathogen and is closely associated with periodontal diseases, a variety of presumably unrelated chronic diseases and multiple cancers [30, 31]. Although the self-reported tooth loss may have a microbial basis in the case of esophageal cancer [16, 17], there is no convincing evidence of direct and specific microbial etiologic agents until our recent findings, which revealed a higher frequency (61%) of *P. gingivalis* presence in ESCC [18].

As *P. gingivalis*is is an important periodontal pathogen in various types of periodontal disease, numerous studies have reported that antibody responses to *P. gingivalis* correlate with severity and progression of periodontitis, extent of attachment loss and treatment effects [32–36]. In a cohort study of NHANES III, not only the increasing severity of periodontitis but the higher serum IgG for *P. gingivalis* was associated with increased orodigestive cancer mortality [25]. In another European prospective cohort study, high levels of antibodies to *P. gingivalis* rendered a > 2-fold increased risk to pancreatic cancer [21]. In clinical settings, serum tumor biomarkers take priority over other measures for screening, diagnosis and clinical management of cancer. However, conventional serum markers for ESCC, such as squamous cell carcinoma antigen (SCCA), carcinoembryonic antigen (CEA), CYFRA21-1 and carbohydrate antigen (CA)19-9, lack sufficient sensitivity and specificity for the early detection and progression of ESCC [37–41].

On the grounds of our recent study establishing the association between the infection of *P. gingivalis* in esophageal epithelium and progression of ESCC, herein we investigate the serum levels of immunoglobulin G and A (IgG and IgA) for *P. gingivalis* and their clinical significance for the diagnosis and postoperative prognosis of ESCC.

## Methods

### Patients

The first cohort of 96 preoperative serum samples were recruited from ESCC patients, who underwent curative esophagectomy at the First Affiliated Hospital of Henan University of Science & Technology and Anyang people’s hospital. None of ESCC patients received preoperative neoadjuvant chemoradiotherapy. The clinical stage of ESCC was classified in accordance with the seventh edition of AJCC and early stage was defined as AJCC stage 0 + I + IIA. Another cohort of 50 serum samples were collected from patients with esophagitis, who underwent gastroscopy. In addition, 80 healthy individuals without evidence of comorbid disease were recruited as healthy controls from the physical examination center of our hospital.

### Enzyme-linked immunosorbent assay

*P. gingivalis* ATCC 33277, used as the antigen in our experiment, was cultured and prepared as previously described. For enzyme-linked immunosorbent assay (ELISA), 100 ul of reconstituted protein extracts of *P. gingivalis* (10 μg/ml) was used to coat microtiter plates followed by incubation with 1:200 diluted serum incubation, 1: 1000 biotin-conjugated anti-human IgG and IgA, and 1:400 avidin-conjugated peroxidase. Antibodies levels were expressed as ELISA units (EUs) with the use of a reference serum pool [42].

### Statistical analysis

The statistical analyses were performed using SPSS 19.0 software package (SPSS, Chicago, IL, USA). Data are expressed as mean ± standard deviation (SD). Comparisons between groups were performed using *t* tests. The receiver operating characteristic (ROC) was used to determine the optimal cut-off value of IgG and IgA. The accuracy, sensitivity, specificity, false negative rate (FNR), false positive rate (FPR) and area under the ROC (AUC) were used to assess the classification efficiency. Overall survival (OS) was defined as the interval between the date of surgery and the date of death or the date of last follow-up. Follow-up data was available for 80 ESCC patients with a median follow-up interval of 10.5 months (3.0-42.6 months). Clinical stage and lymph node metastasis were available for 78 ESCC patients. Survival curves were plotted using the Kaplan-Meier method and differences between curves were tested by log-rank tests. The significance of prognostic factors on survival was studied by Cox regression model.

## Results

### Levels of serum IgG and IgA for *P. gingivalis* in ESCC

**Fig. 1:**
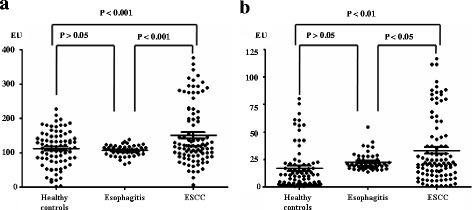
Enzyme-linked immunosorbent assay (ELISA) of serum IgG and IgA antibodies to *P. gingivalis* in healthy controls (*n* = 80), patients with esophagitis (*n* = 50) and ESCC (*n* = 96). **a** Scatter plots of ELISA units (EUs) of *P. gingivalis* IgG antibody in serum of healthy controls, patients with esophagitis and ESCC. **b** Scatter plots of ELISA units (EUs) of *P. gingivalis* IgA antibody in serum of healthy controls, patients with esophagitis and ESCC

The details of ESCC characteristics are presented in Table 1. Figure 1 shows the frequency distributions of IgG and IgA for *P. gingivalis* across the three cohorts. As there were no significant differences between healthy controls and non-ESCC patients with esophagitis with regards to serum levels of *P. gingivalis* IgG or IgA, we combined these two cohorts as non-ESCC controls hereafter. The median serum levels of IgG and IgA for *P. gingivalis* were significantly higher in ESCC patients than in non-ESCC controls (150.69 EU vs. 109.13 EU, *P* < 0.001 for IgG; 33.16 EU vs. 19.14 EU, *P* < 0.01 for IgA). However, no significant correlation was found between serum levels of *P. gingivalis* IgG and IgA (*r*^*2*^ = 0.03, *P* > 0.05, data not shown).

**Table 1:** Associations between serum IgG and IgA antibodies for *P. gingivalis* with clinicopathological features of ESCC

Variables		IgG Titer (EU)	*P*	IgA Titer (EU)	*P*
Age (n(%))	≤ 60(26(32.5%)	132.15 ± 62.13	0.46	31.54 ± 25.93	0.52
	> 60(54(67.5%))	121.07 ± 62.65		38.23 ± 49.45	
Gender (n(%))	Male(55(68.8%))	127.80 ± 62.18	0.51	37.52 ± 48.10	0.65
	Female(25(31.2%))	116.77 ± 63.30		32.84 ± 30.22	
Tobacco use (n(%))	No(43(%))	122.62 ± 58.81	0.75	34.52 ± 30.12	0.73
	Yes(37(%))	127.05 ± 66.87		37.84 ± 55.00	
Alcohol use (n(%))	No(74(%))	125.57 ± 63.66	0.65	61.30 ± 74.42	0.60
	Yes(6(%))	113.47 ± 44.82		54.28 ± 25.41	
Differentiation grade (n(%))	Well(17(21.2%))	166.72 ± 71.77	0.15	36.47 ± 36.07	0.83
	Moderately(47(58.8%))	105.27 ± 45.26		35.12 ± 49.55	
	Poorly(15(18.8))	140.28 ± 72.94		40.17 ± 29.95	
T stage (n(%))	T1 + T2(17(21.5%))	121.72 ± 48.57	0.80	27.17 ± 20.14	0.33
	T3 + T4(62(78.5%))	126.08 ± 66.19		38.89 ± 47.61	
Lymph node metastasis (n(%))	No(44(56.4%))	115.07 ± 57.03	0.19	42.94 ± 57.45	0.27
	Yes(34(43.6%))	134.05 ± 66.21		31.97 ± 28.31	
TNM stage (n(%))	I–II(51(63.8%))	130.18 ± 62.58	0.34	31.77 ± 27.59	0.21
	III–IV(28(36.2%))	115.95 ± 62.60		44.73 ± 62.51

**Fig. 2:**
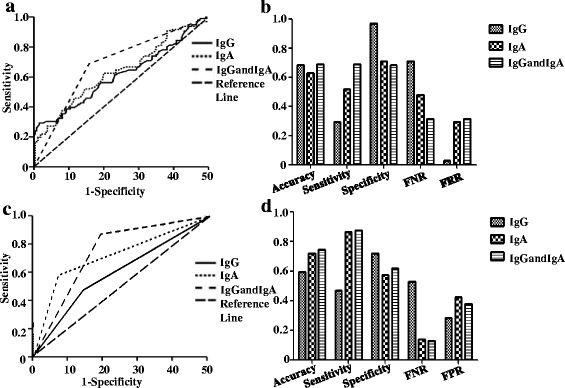
Receiver operating characteristic (ROC) curves and clinical performances of *P. gingivalis* IgG and IgA. **a** ROC curves of IgG, IgA and combination of IgG and IgA for *P. gingivalis* as a diagnostic marker for discrimination of ESCC and non-ESCC controls. **b** Clinical performances of IgG, IgA and combination of IgG and IgA for *P. gingivalis* as a diagnostic marker for discrimination of ESCC and non-ESCC controls in terms of accuracy, sensitivity, specificity, false negative rate (FNR), false positive rate (FPR). **c** Time-dependent ROC curves of IgG, IgA and combination of IgG and IgA for *P. gingivalis* as predictors of ESCC-related 3-year survival rates. **d** Clinical performances of IgG, IgA and combination of IgG and IgA for *P. gingivalis* predictors of ESCC-related 3-year survival rates in terms of accuracy, sensitivity, specificity, false negative rate (FNR), false positive rate (FPR)

Seeking to determine the diagnostic potential of *P. gingivalis* IgG and IgA, ROC curves were plotted to distinguish 96 patients of ESCC from 130 non-ESCC controls. As shown in Fig. 2a, AUCs of IgG and IgA for *P. gingivalis* were 0.612 and 0.632, with optimal cut-off values of 189.17 EU and 21.25 EU, respectively. The specificity for IgG was higher (96.90%) than that of IgA (70.81%) but not the sensitivity (29.17% vs. 52.10%, Fig. 2b). Combination of IgG and IgA, i.e. seropositivity for at least one subtype of IgG or IgA antibody, produced an AUC of 0.686 with a sensitivity of 68.75% and a specificity of 68.46%, respectively (Fig. 2a). Figure 2b shows the diagnostic performance of IgG, IgA, and combination of IgG and IgA in terms of accuracy, sensitivity, specificity, FNR and FPR.

Diagnostic value of IgG and IgA for *P. gingivalis* in early stage of ESCC.

There were 44 patients with early stage disease in our cohort of ESCC. The mean value of *P. gingivalis* IgA in early stage ESCC was lower (32.08 EU) than that of late stage ESCC (41.76 EU) without statistical significance (*P* = 0.29), whereas the mean IgG value was marginally higher in early stage ESCC (114.35 EU vs. 113.62 EU, *P* = 0.058). The sensitivity of *P. gingivalis* IgA for detection of early stage ESCC was 54.54% (24/44) with a specificity of 70.82%, and was far better than that of IgG (20.45%, (9/44)).

### Associations between *P. gingivalis* IgG and IgA with clinicopathological features and overall survival of ESCC

The associations between clinicopathological features of ESCC and serum levels of IgG or IgA for *P. gingivalis* were determined by *t* test. No significant associations were observed between any clinicopathological features with IgG or IgA serum levels. Likewise, ROCs were plotted to predict the 3-year OS rate of ESCC. Figure 2c shows the time-dependent ROC curves of *P. gingivalis* antibodies as predictors of ESCC-related 3-year survival rates and the AUCs were 0.595 and 0.719 with optimal cut-off values of 125.08 EU and 37.12 EU for IgG and IgA, respectively. The sensitivity of *P. gingivalis* IgA was higher than that of IgG (86.25% vs. 47.82%) but not the specificity (57.54% vs. 71.92%, Fig. 2d). Likewise, combination of IgG and IgA produced a maximal AUC (0.746), a maximal sensitivity (87.16%) but a modest specificity (62.07%) in comparison with individual IgG or IgA (Fig. 2d).

**Fig. 3:**
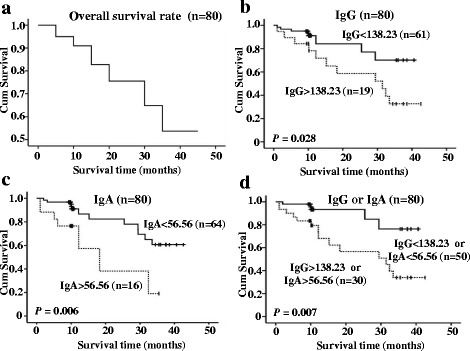
Kaplan-Meier survival curves of ESCC patients. **a** The 3-year OS rate of 80 ESCC patients was 52.23%. **b** The 3-year OS rates in ESCC patients with IgG < 138.23 EU (*n* = 61) and IgG > 138.23 EU (*n* = 19) were 70.145% and 32.68%, respectively, with a significant difference (*P* = 0.028). **c** The 3-year OS rates in ESCC patients with IgA < 56.56 EU (*n* = 64) and IgG > 56.56 EU (*n* = 16) were 60.82% and 18.83%, respectively, with a significant difference (*P* = 0.006). **d** The 3-year OS rates in ESCC patients with IgG < 138.23 EU or IgA < 56.56 (*n* = 50) and IgG > 138.23 EU or IgA > 56.56 (*n* = 30) were 76.38% and 34.04%, respectively, with a significant difference (*P* = 0.007)

Figure 3a shows the postoperative survival of 80 ESCC patients with a median survival time of 31.58 months, 61 surviving patients and 19 ESCC-related deaths at the last clinical follow-up (Fig. 3a). Using the optimal cut-off value of 138.23 EU, Kaplan-Meier survival analysis revealed that ESCC patients with higher serum level of *P. gingivalis* IgG had a significantly worse prognosis than ESCC with lower serum level (log-rank test, x^2^ = 4.852, *P* = 0.028, median OS of 26.25 (*n* = 19) months vs. 33.68 months (*n* = 61), Fig. 3b). The prognostic effect of *P. gingivalis* IgA resembled that of IgG (log-rank test, x^2^ = 6.800, *P* = 0.006, median OS of 19.59 months (*n* = 16) vs. 34.15 months (*n* = 64), Fig. 3c). In 50 ESCC patients with lower IgG or IgA serum level, the median OS was 36.12 months compared with 25.89 months of their counterparts (log-rank test, x^2^ = 7.208, *P* = 0.007, Fig. 3d). Furthermore, 5 ESCC patients with higher levels of both IgG and IgA had the worst prognosis and the median OS for these 5 patients was 16.62 months versus 32.93 months of the other 75 patients (log-rank test, x^2^ = 8316, *P* = 0.004, Data now shown).

The prognostic values of histopathological features were also evaluated by Kaplan-Meier method and log-rank test. With regards to clinical TNM stage, stage I–II ESCC patients (stage I–II, 63.75%, *n* = 51) survived longer than stage III–IV ESCC cases (Stage III–IV, 36.25%, *n* = 27, Additional file 1: Figure S1A). For the subgroup ESCC patients with early clinical stage, a significant benefit in OS was observed in patients with low serum level of *P. gingivalis* IgA but non-significant for IgG than in patients with high level (log-rank test, x^2^ = 9.141, *P* = 0.003, Additional file 1: Figure S1B & D), and neither IgG nor IgA was associated with OS of late stage ESCC (Additional file 1: Figure S1C &E). In addition, lymph node metastasis was significantly associated with shorter OS ((log-rank test, x^2^ = 5.61, *P* = 0.018, Additional file 2: Figure S2A). In ESCC patients with negative lymph node metastasis, those with high levels of *P. gingivalis* IgG or IgA had worse OS than patients with low IgG or IgA serum level (log-rank test, x2 = 6.097/6.097, *P* = 0.014/0.011, Additional file 2: Figure S2B & D), whereas no significant differences were observed between *P. gingivalis* IgG or IgA and OS in positive lymph node metastasis (Additional file 2: Figure S2C & E).

To identify independent prognostic factors for ESCC patients, clinicopathological factors were assessed by univariate and multivariate Cox regression models. Univariate Cox proportional hazard regression analysis revealed that N-stage (Hazard ratio = 3.169, 95% CI = 1.175 – 8.545, *P* = 0.023), IgG (Hazard ratio = 3.039, 95% CI = 1.148 – 8.041, *P* = 0.025) and IgA (Hazard ratio = 3.588, 95% CI = 1.368 – 9.409, *P* = 0.009) were significant prognostic predictors for OS of ESCC patients (Table 2). When N-stage, IgG and IgA were analysed by multivariate analysis using Cox’s proportional hazards model, N-stage (Hazard ratio = 12.292, 95% CI = 1.399 – 108.003, *P* = 0.024), IgG (Hazard ratio = 4.910, 95% CI = 1.473– 16.364, *P* = 0.010) and IgA (Hazard ratio = 4.686, 95% CI = 1.492 – 14.722, *P* = 0.008) were independent prognostic factors of ESCC (Table 2).

**Table 2:** Univariate and multivariate Cox regression analyses of the prognostic variables in ESCC patients

Variables	Subsets	Univariate analysis (*n* = 79)			Multivariate analysis (*n* = 79)		
		Hazard ratio	95% CI	*P*	Hazard ratio	95% CI	*P*
Age	≤ 60 vs. >60	1.779	0.628–5.042	0.278	–	–	–
Gender	Male vs. Female	0.601	0.212–1.703	0.338	–	–	–
T-stage	T1 + T2 vs. T3 + T4	0.875	0.252–3.037	0.834	–	–	–
N-stage	No vs. Yes	3.169	1.175–8.545	0.023	12.292	1.399–108.003	0.024
Histological grade	G1 vs. G2–G3	2.470	0.920–6.631	0.073	–	–	–
Clinical stage	I–II vs. III–IV	0.407	0.150–1.104	0.077	–	–	–
IgG (EU)	≤ 2760 vs. >2760	3.039	1.148–8.041	0.025	4.910	1.473–16.364	0.010
IgA (EU)	≤ 1130 vs. >1130	3.588	1.368–9.409	0.009	4.686	1.492–14.722	0.008

## Discussion

Early diagnosis remains one of the key determinants to improve the long-term survival of patients with ESCC. The majority of patients with ESCC present at an advanced stage and have limited treatment options, resulting in dismal prognosis [1, 3–7]. Although gastroscopy with biopsy offers an efficient method for diagnosis of patients with ESCC, poor compliance of gastroscopy in asymptomatic patients precludes early detection. Compared with gastroscopy, blood testing is less invasive and cost-effective. Therefore, serum biomarkers have the priority over other measures for clinical application to detect ESCC at an early stage [43].

First and foremost, the present study demonstrates that serum antibody levels against *P. gingivalis* have the potential for diagnosis of ESCC. Although inflammation plays a key role in esophageal carcinogenesis, our results revealed that morphological esophagitis harboring inflammatory cells without transformed cells in esophageal mucosa failed to show increased IgG and IgA antibody response to *P. gingivalis*. This finding indicates that *P. gingivalis* may not be involved in the process of esophagitis, but do not rule out the possibility that *P. gingivalis* or host responses against *P. gingivalis* contribute to the development and progression of ESCC. In sharp contrast, titers of IgG and IgA against *P. gingivalis* in serum of patients with ESCC increased remarkably compared to patients with esophagitis and healthy controls, which provides direct evidence that *P. gingivalis* is implicated in the pathogenesis of ESCC. Using an optimal diagnostic cut off value of 425 EU, individual IgA had the highest sensitivity (52.1%) for discrimination of ESCC from non-ESCC controls compared with conventional serum markers for ESCC, such as SCCA, CYFRA21-1, CEA, CA19-9 [37–41], whereas the specificity was low (70.8%). However, ELISA results of SCCA1, SCCA2, CYFRA21-1 and CEA did not show diagnostic value in our cohort (data not shown). Growing evidence indicates that combination of several individual biomarkers is superior to any single biomarker [44]. Combination of IgG and IgA for *P. gingivalis* had an increased AUC (0.671) compared with an individual IgG or IgA.

For detection of early stage ESCC, conventional serum biomarkers of ESCC have little diagnostic benefit. For instance, the positive frequencies of both CYFRA21-1 and SCCA in patients with early stage ESCC (stage 0-II) varied from 4.7% to 24% [37, 40]. In contrast, the diagnostic performance of serum *P. gingivalis* IgA for early ESCC was superior as evidenced by a sensitivity of 54.54% in our study. Although the specificity of single IgA was not sufficient, combination of IgG and IgA produced a specificity of 91.5%.

Mounting clinical evidence indicates a positive association between *P. gingivalis* or periodontal disease and an increased risk for a variety of cancers and even poor prognosis [11, 12, 18, 21, 25]. In normal distal esophagus, bacterial colonization was not uncommon [45]. Furthermore, the global esophageal microbiome in both esophagitis and Barrett’s esophagus altered from typeI bacteria in normal esophageal mucosa to typeIIbacteria, many of which are Gram-negative anaerobes/microaerophiles and putative pathogens of periodontal disease [46]. Our previous study demonstrated that *P. gingivalis* infection in ESCC was prevalent (61%) and negatively correlated with OS of ESCC [18]. In the present study, we looked into the prognostic potential of human immune response to *P. gingivalis* in terms of IgG and IgA. In line with the presence of *P. gingivalis* in ESCC, higher serum levels of *P. gingivalis* IgG and IgA were associated with worse prognosis of patients with ESCC. In particular for early stage ESCC, i.e. ESCC with stage 0-II or negative lymphnode metastasis, patients with high level of *P. gingivalis* IgG or IgA had a significantly lower OS relative to ESCC patients with low level, and patients with high level of both IgG and IgA had the worst prognosis. Multivariate analysis identified lymph node status, IgG and IgA as independent prognostic factors. Therefore, IgG and IgA were combined and we found that the combination produced higher predictive accuracy than an individual IgG or IgA.

## Conclusions

To our knowledge, we are the first to report that the human immune response against *P. gingivalis* is implicated in the malignant progression of ESCC. IgG and IgA for *P. gingivalis* are potential serum biomarkers for ESCC and combination of IgG and IgA improves the diagnostic and prognostic performance. Furthermore, serum IgG and IgA for *P. gingivalis* could differentiate early stage ESCC patients. Further investigations are warranted to compare or combine with current serum biomarkers for ESCC, to identify the optimal panel for clinical application.

## Additional files

Additional file 1: Figure S1. Kaplan-Meier survival curves of ESCC patients with regards to clinical stage. A The 3-year OS rates in ESCC patients with TNMI-II (*n* = 51) and patients with TNM III-IV (*n* = 27) were 59.95% and 33.26%, respectively (*P* = 0.069). B The 3-year OS rates in ESCC patients with IgG < 138.23 EU (*n* = 59) and IgG > 138.23 EU (*n* = 19) were 77.59% and 37.65%, respectively, in early clinical stage (*P* = 0.055). B The 3-year OS rates in ESCC patients with IgG < 138.23 EU (*n* = 59) and IgG > 138.23 EU (*n* = 19) were 44.63% and 20.89%, respectively, in late clinical stage (*P* = 0.055). D The 3-year OS rates in ESCC patients with IgA < 56.56 EU (*n* = 62) and IgA > 56.56 EU (*n* = 16) were 68.95% and 23.34%, respectively, in early clinical stage (*P* = 0.003). D The 3-year OS rates in ESCC patients with IgA < 56.56 EU (*n* = 62) and IgA > 56.56 EU (*n* = 16) were 41.45% and 0, respectively, in late clinical stage (*P* = 0.48). (DOC 334 kb)

Additional file 2: Figure S2. Kaplan-Meier survival curves of ESCC patients with regards to lymph node stage. A The 3-year OS rates in ESCC patients without lymph node metastasis (*n* = 44) and patients with lymph node metastasis (*n* = 34) were 63.87% and 27.85%, respectively (*P* = 0.018). B The 3-year OS rates in ESCC patients with IgG < 138.23 EU (*n* = 59) and IgG > 138.23 EU (*n* = 19) were 87.19% and 37.64%, respectively, in negative lymph node metastasis (*P* = 0.014). C The 3-year OS rates in ESCC patients with IgG < 138.23 EU (*n* = 59) and IgG > 138.23 EU (*n* = 19) were 29.43% and 20.80%, respectively, in lymph node metastasis (*P* = 0.293). D The 3-year OS rates in ESCC patients with IgA < 56.56 EU (*n* = 62) and IgA > 56.56 EU (*n* = 16) were 72.91% and 25.96%, respectively, in negative lymph node metastasis (*P* = 0.011). E The 3-year OS rates in ESCC patients with IgA < 56.56 EU (*n* = 62) and IgA > 56.56 EU (*n* = 16) were 34.52% and 0, respectively, in lymph node metastasis (*P* = 0.092). (DOC 355 kb)

## Abbreviations

AUC: Area under the ROC curve
ESCC: Esophageal squamous cell carcinoma
IgG/A: Immunoglobulin G/A
ROC: Receiving operating characteristic
