# Supplementary material for: Preoperative serum immunoglobulin G and A antibodies to Porphyromonas gingivalis are potential serum biomarkers for the diagnosis and prognosis of esophageal squamous cell carcinoma
Source: BMC Cancer. 2018 Jan 3;18:17. doi: 10.1186/s12885-017-3905-1 (PMC5753462; doi:10.1186/s12885-017-3905-1)

**Additional file 1: Figure S1. Kaplan-Meier survival curves of ESCC patients with**

**regards to clinical stage.**


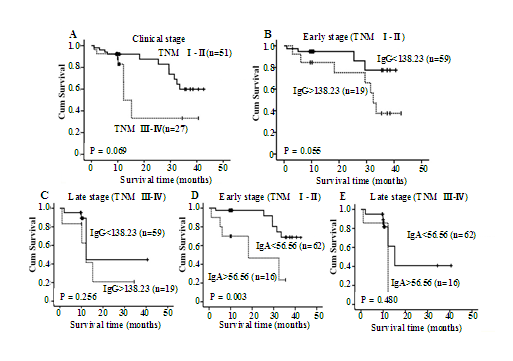

Supplement: Supplementary file 1 — Kaplan-Meier survival curves of ESCC patients with regards to clinical stage. A The 3-year OS rates in ESCC patients with TNMI-II (n = 51) and patients with TNM III-IV (n = 27) were 59.95% and 33.26%, respectively (P = 0.069). B The 3-year OS rates in ESCC patients with IgG < 138.23 EU (n = 59) and IgG > 138.23 EU (n = 19) were 77.59% and 37.65%, respectively, in early clinical stage (P = 0.055). B The 3-year OS rates in ESCC patients with IgG < 138.23 EU (n = 59) and IgG > 138.23 EU (n = 19) were 44.63% and 20.89%, respectively, in late clinical stage (P = 0.055). D The 3-year OS rates in ESCC patients with IgA < 56.56 EU (n = 62) and IgA > 56.56 EU (n = 16) were 68.95% and 23.34%, respectively, in early clinical stage (P = 0.003). D The 3-year OS rates in ESCC patients with IgA < 56.56 EU (n = 62) and IgA > 56.56 EU (n = 16) were 41.45% and 0, respectively, in late clinical stage (P = 0.48). (DOC 334 kb) [file 12885_2017_3905_MOESM1_ESM.doc]
