# Supplementary material for: Preoperative serum immunoglobulin G and A antibodies to Porphyromonas gingivalis are potential serum biomarkers for the diagnosis and prognosis of esophageal squamous cell carcinoma
Source: BMC Cancer. 2018 Jan 3;18:17. doi: 10.1186/s12885-017-3905-1 (PMC5753462; doi:10.1186/s12885-017-3905-1)

**Additional file 2: Figure S2. Kaplan-Meier survival curves of ESCC patients with regards to lymph node stage.**


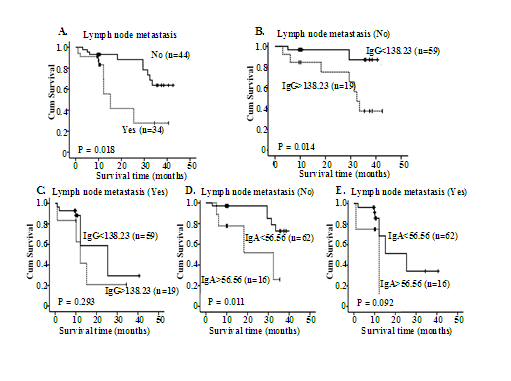

Supplement: Supplementary file 2 — Kaplan-Meier survival curves of ESCC patients with regards to lymph node stage. A The 3-year OS rates in ESCC patients without lymph node metastasis (n = 44) and patients with lymph node metastasis (n = 34) were 63.87% and 27.85%, respectively (P = 0.018). B The 3-year OS rates in ESCC patients with IgG < 138.23 EU (n = 59) and IgG > 138.23 EU (n = 19) were 87.19% and 37.64%, respectively, in negative lymph node metastasis (P = 0.014). C The 3-year OS rates in ESCC patients with IgG < 138.23 EU (n = 59) and IgG > 138.23 EU (n = 19) were 29.43% and 20.80%, respectively, in lymph node metastasis (P = 0.293). D The 3-year OS rates in ESCC patients with IgA < 56.56 EU (n = 62) and IgA > 56.56 EU (n = 16) were 72.91% and 25.96%, respectively, in negative lymph node metastasis (P = 0.011). E The 3-year OS rates in ESCC patients with IgA < 56.56 EU (n = 62) and IgA > 56.56 EU (n = 16) were 34.52% and 0, respectively, in lymph node metastasis (P = 0.092). (DOC 355 kb) [file 12885_2017_3905_MOESM2_ESM.doc]
